# PTIR: Predicted Tomato Interactome Resource

**DOI:** 10.1038/srep25047

**Published:** 2016-04-28

**Authors:** Junyang Yue, Wei Xu, Rongjun Ban, Shengxiong Huang, Min Miao, Xiaofeng Tang, Guoqing Liu, Yongsheng Liu

**Affiliations:** 1 School of Biotechnology and Food Engineering, Hefei University of Technology, Hefei 230009, China; 2 School of Information Science and Technology, University of Science and Technology of China, Hefei 230026, China; 3 Ministry of Education Key Laboratory for Bio-resource and Eco-environment, College of Life Science, State Key Laboratory of Hydraulics and Mountain River Engineering, Sichuan University, Chengdu 610064, China

## Abstract

Protein-protein interactions (PPIs) are involved in almost all biological processes and form the basis of the entire interactomics systems of living organisms. Identification and characterization of these interactions are fundamental to elucidating the molecular mechanisms of signal transduction and metabolic pathways at both the cellular and systemic levels. Although a number of experimental and computational studies have been performed on model organisms, the studies exploring and investigating PPIs in tomatoes remain lacking. Here, we developed a Predicted Tomato Interactome Resource (PTIR), based on experimentally determined orthologous interactions in six model organisms. The reliability of individual PPIs was also evaluated by shared gene ontology (GO) terms, co-evolution, co-expression, co-localization and available domain-domain interactions (DDIs). Currently, the PTIR covers 357,946 non-redundant PPIs among 10,626 proteins, including 12,291 high-confidence, 226,553 medium-confidence, and 119,102 low-confidence interactions. These interactions are expected to cover 30.6% of the entire tomato proteome and possess a reasonable distribution. In addition, ten randomly selected PPIs were verified using yeast two-hybrid (Y2H) screening or a bimolecular fluorescence complementation (BiFC) assay. The PTIR was constructed and implemented as a dedicated database and is available at http://bdg.hfut.edu.cn/ptir/index.html without registration.

The increasing number of complete genome sequences has revealed the entire structure and composition of proteins, based mainly on theoretical predictions utilizing their corresponding DNA sequences. Although proteins are essential parts of organisms and participate in virtually every process within a cell, this annotation is only mapped in one dimension. In fact, proteins, as vital macromolecules, rarely act alone. At both the cellular and systemic levels, almost all the molecular processes involve a large number of protein-protein interactions (PPIs). Consequently, PPIs form a two-dimensional network to perform complex cellular functions and relay information between the environment, the cell and the genome^1^. When identified on a genome-wide scale, PPIs are commonly visualized as protein interaction networks (PINs), which are also known as interactomes^2^. The increasing number of interactome studies has greatly expanded the flexibility of proteins beyond their individual activities. Therefore, deciphering the PINs could facilitate understanding the molecular basis of the interactions and the complex biological phenotypes^3^.

Many efforts have been made to chart PPIs. In several model organisms, including *Arabidopsis thaliana*^4^,^5^, *Caenorhabditis elegans*^6^,^7^, *Drosophila melanogaster*^8^,^9^, *Homo sapiens*^10^,^11^,^12^, and *Saccharomyces cerevisiae*^13^,^14^, genome-wide yeast two-hybrid (Y2H) systems and large-scale affinity purification/mass spectrometry (MS) studies have been conducted to map the interactomes. Meanwhile, certain databases, such as IntAct^15^, BioGRID^16^ and DIP^17^, have been established as repositories to collect and organize the reported protein interactions of nonspecific species. Despite these improved protocols and evolved methods, the cost and time requirements of such exploratory experimental studies remain prohibitive, and thus only small to mid-sized PIN studies have been conducted^18^,^19^. Alternatively, proteomic studies are progressively shifting away from classical approaches that focus on a few proteins toward whole PINs to chart the complex and dynamic interactions in cellular processes. As a result, bioinformatics approaches are desirably employed as a valuable preliminary step to identify potential protein interactions^20^,^21^. Using computational methods based on protein phylogenetic distances, a series of PPIs have been predicted to increase the number of available protein interaction datasets, such as the STRING database^22^. However, a limited number of plant PPIs have been included in these databases so far (Table 1). In addition, the progress of PPI predictions in single plant species is rather slow, with only *Arabidopsis thaliana*^23^,^24^,^25^, *Oryza sativa*^26^, *Brassica rapa*^27^, *Zea mays*^28^ and *Populus trichocarpa*^29^ being reported to date.

While interologs-based approaches for predicting protein interactions have been successfully developed and applied to many species^23^,^26^,^27^,^30^,^31^,^32^, the introduction of large-scale experimental interactome approaches would provide new opportunities to predict protein interactions using machine learning algorithms that tend to improve the prediction accuracy when training datasets containing larger numbers and greater diversity are used^29^,^33^,^34^. Additionally, several attempts have been made to develop evolving approaches based on PIN topology^25^,^35^,^36^. Although these topology-based approaches possess an apparent advantage of simplicity, they have difficulty in identifying the PPIs associated with protein complexes^37^. Overall, the distinct approaches employed by different researchers have provided unique but incomplete network information^29^. Therefore, diverse approaches using multiple features are often comprehensively incorporated to increase prediction confidence.

Considering the scale of experimental PPI data and the high risk of error propagation, we have constructed a predicted tomato interactome by identifying potential PPIs from interacting orthologs in Arabidopsis (*Arabidopsis thaliana*), nematode worm (*Caenorhabditis elegans*), fruit fly (*Drosophila melanogaster*), human (*Homo sapiens*), rice (*Oryza sativa*), and yeast (*Saccharomyces cerevisiae*). To date, we have obtained 357,946 non-redundant tomato PPIs (integrated with 12 additional experimentally reported PPIs in the IntAct database) among 10,626 proteins. These interactions are expected to cover 30.6% of the entire tomato proteome and possess a high level of accuracy. To facilitate further research, we have developed and characterized a searchable database called the Predicted Tomato Interactome Resource (PTIR). The PTIR features a user-friendly interface that allows individuals to search the database, browse the information and visualize the data. This resource and the related documents are freely accessible at http://bdg.hfut.edu.cn/ptir/index.html.

## Results

### Building the interactome and data statistics

PPIs, which play central roles in signal transduction and metabolic pathways, were predicted based on the assumption that evolutionarily conserved proteins would be likely to exhibit conserved interactions. This process is known as interaction ortholog mapping and served as an established method for predicting interactomes^38^. Over the years, it has been successfully applied in human^30^, yeast^31^, Arabidopsis^23^,^24^, rice^26^ and *Brassica rapa*^27^. Here, we constructed a tomato interactome by referring to experimental PPI datasets from in-depth studies of six model organisms: Arabidopsis, nematode worm, fruit fly, human, rice and yeast. Among these species, Arabidopsis shares the highest evolutionary conservation with tomatoes, whereas yeast has the best coverage of its genome. Subsequently, the potential tomato PPIs were identified where orthologous protein groups of both interactive members existed in any one of these six established interactomes. Application of this method resulted in 357,946 predicted interactions among 10,626 tomato proteins. Of these, 3,289 were predicted as self-interactions (homodimers) and 354,657 were interactions between different proteins (heterodimers).

**Figure 1:**
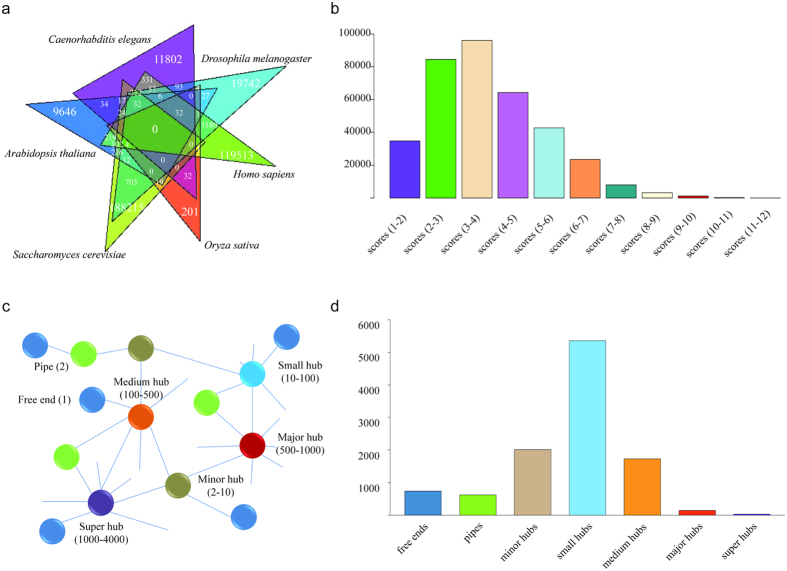
(**a**) Contributions of the six species to our predicted tomato interactome. (**b**) Frequency distribution of the statistical scores. (**c**) Different types of protein nodes classified according to the interacting partners. (**d**) Frequency distribution of the different node types.

We then mapped the predicted tomato PPIs to these six species’ interactomes and determined the number of PPIs deduced from one specific species; the numbers of PPIs common to two, three, four, or five species; and the number of PPIs conserved in all six species (Fig. 1a). As Fig. 1a shows, no protein interactions were identified from all six species, and only 8,827 protein interactions were predicted from more than one species, corresponding to approximately 2.5% of the whole predicted tomato interactome. This value is similar to that in rice (3.7%)^26^. The poor overlap among the datasets may be because of the relatively incomplete nature of experimentally derived interactomes in different species.

The predicted tomato PPIs were generated through inter-species comparisons. The confidence of these datasets must be evaluated. Therefore, we treated all the identified PPIs as repertoires to derive other parameters that allow for the scoring of each protein pair. It is based mainly on the following: (1) the number of species (six species in total) the interactions were predicted from, (2) the number of species (six species in total) in which the two interacting proteins evolved together, (3) the number of gene ontology (GO) terms (out of three) shared by each pair of interacting proteins, (4) whether the domains of the proteins have the potential to interact, (5) whether the subcellular localization of the proteins are available for interaction, and (6) whether the protein pairs are co-expressed. Based on these assessments, 12,291 high-confidence interactions (total scores >=7), 226,553 medium-confidence interactions (total scores between 2 and 7), and 119,102 low-confidence interactions (total scores <2) were identified. The logarithmic distributions of the more elaborate statistical scores (between 1 and 12) are displayed in Fig. 1b.

Next, interacting proteins of the predicted tomato interactome along with their connections were loaded into the network-building program CYTOSCAPE^39^ to visualize the composition and topology. Surprisingly, 10,500 out of 10,626 conserved proteins were connected into a single interconnected network, whereas the remaining 126 proteins were organized by only several connections (one to nine). In the core-interconnected network, many proteins had a high number of interacting partners, including those involved in protein folding (e.g., members of the heat shock protein and DNAJ chaperone protein families) and protein synthesis (e.g., elongation factor 1-alpha) (Supplementary Table S1). Previous studies have showed that these proteins have fundamental cellular functions and belong to an ancient protein family^40^,^41^.

**Figure 2:**
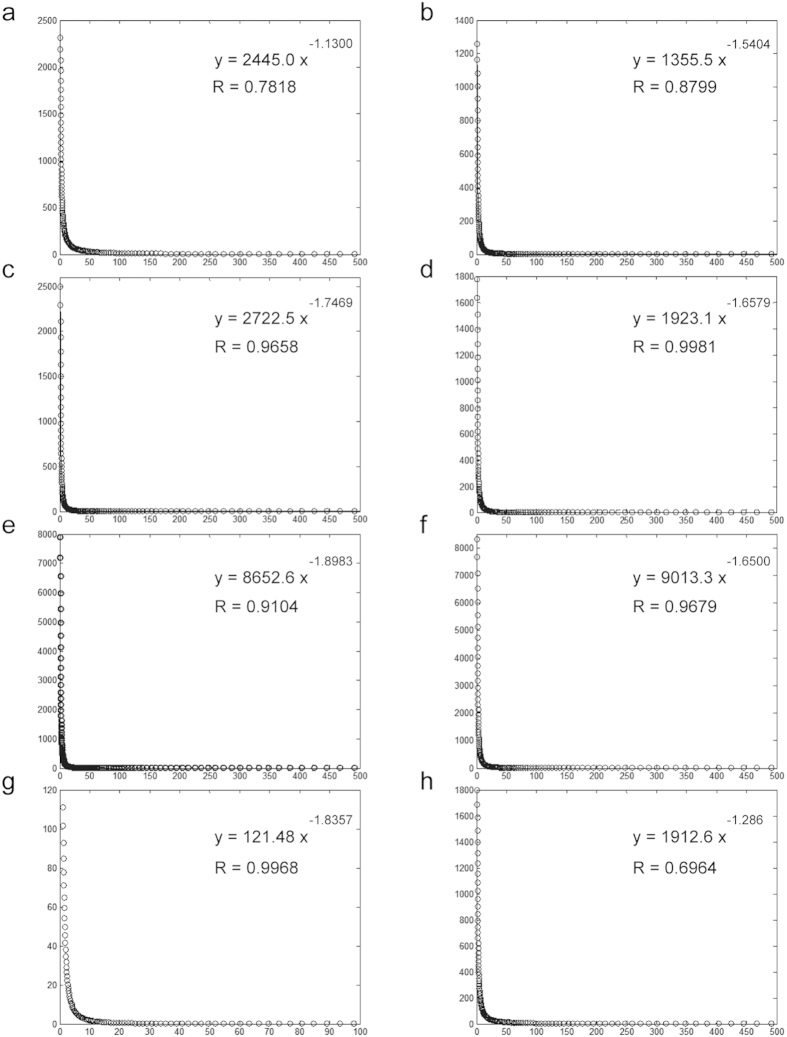
The hub connectivity follows a scale-free power law distribution. (**a**) Data in the PTIR. (**b**) The High_quality_0.6 dataset. (**c**) Arabidopsis interactome. (**d**) Nematode worm interactome. (**e**) Fruit fly interactome. (**f**) Human interactome. (**g**) Rice interactome. (**h**) Yeast interactome.

To further analyze the topology of the interaction network, proteins were divided into free ends (with only one interaction), pipes (two interactions), and hubs (multiple interactions) of different sizes (minor hubs, small hubs, medium hubs, major hubs, and super hubs) (Fig. 1c). As displayed in Fig. 1d, the hub distribution shows that more than half of the proteins belong to small hubs with interactions between 10 and 100 neighbors. Compared with hub connectivity of these reference species, the distribution details vary slightly because of the different grouping standard but always follow a scale-free power law distribution (Fig. 2). This phenomenon has been observed in other studies^23^,^42^. Because the categories of hub connectivity are directly associated with the number of interacting proteins, their size shifts as the individual interactomes grow. In the current study, the interacting proteins possess, on average, 35 neighbors, more than in Arabidopsis and rice but similar to *Brassica rapa* (Table 2). These comparisons also indicate that the average number of interacting partners will increase as the interactome coverage increases, implying that our predicted interactome has relatively good coverage.

**Figure 3:**
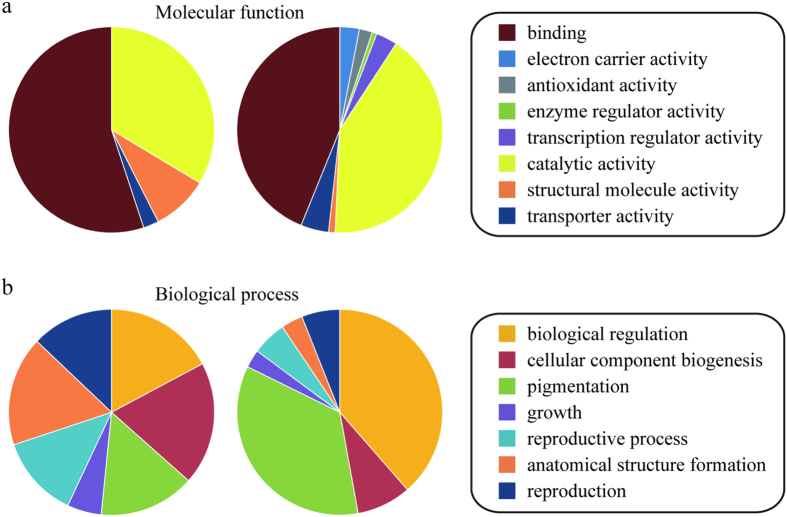
The GO annotation of the proteins in the large hubs (left) and free ends (right). (a) The molecular function category. (**b**) The biological process category.

Additionally, the proteins in large hubs (including major and super hubs) and free ends were assigned to molecular function and biological process categories according to the GO annotation from the Gene Ontology Consortium (Supplementary Tables S2 and S3)^43^. This suggests that proteins in large hubs are significantly enriched for binding and structural molecule activity, whereas proteins in free ends tend to possess electron carrier activity and antioxidant activity (Fig. 3a). As expected, most proteins in large hubs fall into the essential biological processes categories, including cellular component biogenesis, growth, anatomical structure formation, response to stimulus, and reproduction (Fig. 3b). This enrichment indicates that proteins with more interacting partners are likely to be more essential^44^. Similarly, essential proteins and complexes are also likely to have a relatively large number of neighbors in the PIN^45^. However, the large number only suggests a potential capacity of these tremendous interactions, and the actual connectivity of such interactions in a given cell or tissue depends on the differential expression of genes.

### Quality control of the PTIR

The initial interaction datasets used for our prediction were downloaded from the IntAct database. Although these interactions were characterized experimentally, they were originally generated from a range of different approaches, such as various experimental detection methods, observed evidence, and interaction types^15^. To systematically evaluate and compare the assessments of the individual interactions, IntAct implemented the MI-score, which is a confidence score based on common and minimum curated information^15^. By applying different thresholds for the MI-score, we obtained a series of rigorous or tolerant datasets based on orthologous predictions (Table 3). Although using a higher standard increases the confidence of each dataset, it is possible exclude a large number of possible protein interactions. Conversely, a lower standard may help to recover additional plausible interactions at the expense of including more false information. Collectively, we calculated the frequency and enrichment of high-confidence interactions for all the “high-quality datasets”. As shown in Table 3, the frequency and enrichment of high-confidence interactions increased as the MI-score increased, providing strong evidence of the effectiveness of applying a higher threshold.

### The co-evolution of the interacting proteins

**Figure 4:**
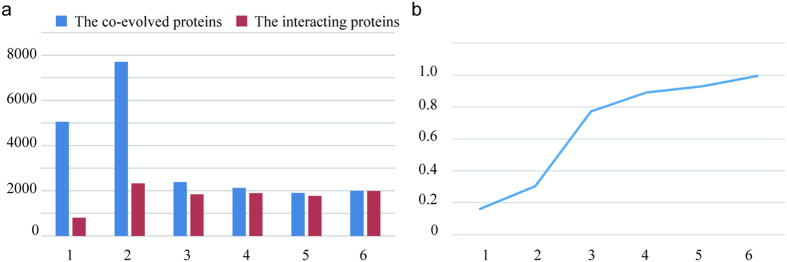
The statistical analysis of co-evolving proteins. (**a**) The number of orthologous proteins and interacting proteins identified in the PTIR across the various species. (**b**) The proportion of interacting proteins and orthologous proteins across the various species.

Interacting protein pairs often co-evolve because they need to perform a given function together or disappear from evolution because they cannot work individually^46^. Recently, the extreme phenomenon of the presence/absence of co-evolving orthologs has been used as the basis for the “phylogenetic profiling” method used to detect potential interacting proteins^47^,^48^. Therefore, investigating the characteristics of co-evolution could improve the effective scope of protein interaction predictions. In tomatoes, 21,160 proteins have orthologs in at least one of the six reference species. Of these, 10,626 proteins were identified as interacting proteins/partners in the PTIR. Subsequently, we analyzed the number of co-evolved proteins and those included in the PTIR for the different reference species. As shown in Fig. 4a, almost all the co-evolved proteins among the six species were identified as having interactions in the PTIR. By contrast, only a small number of the co-evolved proteins existing in only one species were involved in the predicted PPIs. Therefore, the more two proteins interact with each other, the more likely it is that they co-evolved, suggesting that it is possible to make inferences about interactions between co-evolved proteins based on their phylogenetic profiling (Fig. 4b).

### Evolutionary conservation of domain-domain interactions

Domains are the main functional and structural units of proteins^49^. They often play a crucial role in PPIs by binding in diverse combinations (heterotypic or homotypic). Because the assignment of interologs is based on global protein sequence similarity, these domains may be evolutionarily maintained across species^50^. Generally, domain-domain interactions (DDIs), which are the building blocks of PPIs, are more conserved than PPIs^51^. Here, we used the DDIs of all the proteins to validate and examine the probabilities of our predicted tomato PPIs at the domain level. The DDI datasets were taken from the Database of Protein Domain Interactions (DOMINE)^52^, which contains both experimentally observed and computationally predicted DDIs. Each protein domain was assigned a Pfam identity using the HMMER algorithm^53^. In total, we identified 2,806 unique Pfam domains among the 10,169 predicted tomato proteins (approximately 95.7% of the total predicted tomato proteins). On average, 1.42 (15057/10626) domains were assigned to each tomato protein. This value is comparable with those of Pfam annotation in *Arabidopsis thaliana*^54^ and *Brassica rapa*^27^ (1.41 and 1.43 domains/proteins, respectively). As domains are shared by various interacting proteins and because different PPIs could be mediated by the same domain pairs, 110,609 PPIs were determined based on the DDIs. This finding will not only increase the confidence of our predicted tomato interactome, but also provide more detailed information regarding the domains that are potentially involved in mediating protein interactions. The remaining interacting protein pairs without DDI assignment exist because no domain assignment can be made yet or they are mediated by short motifs, which may form transient rather than stable interactions^55^.

### Subcellular localization of interacting proteins

Subcellular localization is a process by which proteins are targeted to a specific location within a cell, such as the nucleus, cytoplasm or cell membrane. During interactions, the interacting proteins are generally co-localized in the same subcellular location^56^. Analyzing the subcellular localization of interacting proteins improves the reliability of the predicted PPIs. Currently, no specific subcellular localization database exists for tomatoes. To assign the subcellular localization of the proteins in our predicted tomato interactome, we organized the related data from the UniProt database^57^ and made predictions for the rest of the proteins using TargetP software^58^. In total, we obtained 208,351 protein interactions with subcellular localization information for 9,244 unique proteins. Using these data, we searched for PPIs whose interacting partners were co-localized in the same subcellular location or available compartments. A total of 86,778 PPIs were confirmed by co-localization analysis^59^. This number accounts for 41.65% of the entire interactome, slightly less than in the rice interactome that (49.1%)^26^.

### The co-expression of interacting proteins

Proteins that exhibit interactions may display similar dynamic or static patterns of gene expression under various experimental conditions^60^. Consequently, if the expression of interacting protein pairs synchronously rises or falls, the possibility of PPIs between them might be greatly increased. Therefore, an assessment of the co-expression of two proteins strengthens the confidence regarding the prediction of their interaction^61^. However, the lack of gene expression correlation does not necessarily mean that the two proteins do not interact. Conversely, it could suggest that one partner is constitutively expressed, whereas the other is expressed under certain conditions or in specific tissues.

In our study, protein co-expression was calculated by applying the Pearson correlation coefficient (*r*) to the expression data for each PPI of the predicted tomato interactome (see the Methods section). Each interaction was given a co-expression score (CS) for the possibility of protein interactions. A total of 349,794 PPIs in our predicted tomato interactome were identified using the CS value, and only 8,152 (approximately 2.3%) interactions had no expression information in all 96 Gene Expression Omnibus (GEO) samples (Supplementary Table S4). This measurement was used as a reference for the confidence of the predicted PPIs. Additionally, proteins with unknown functions that were co-expressed with known proteins were assumed to be involved in the same biological process.

### Y2H studies and biomolecular fluorescence complementation analysis

**Figure 5:**
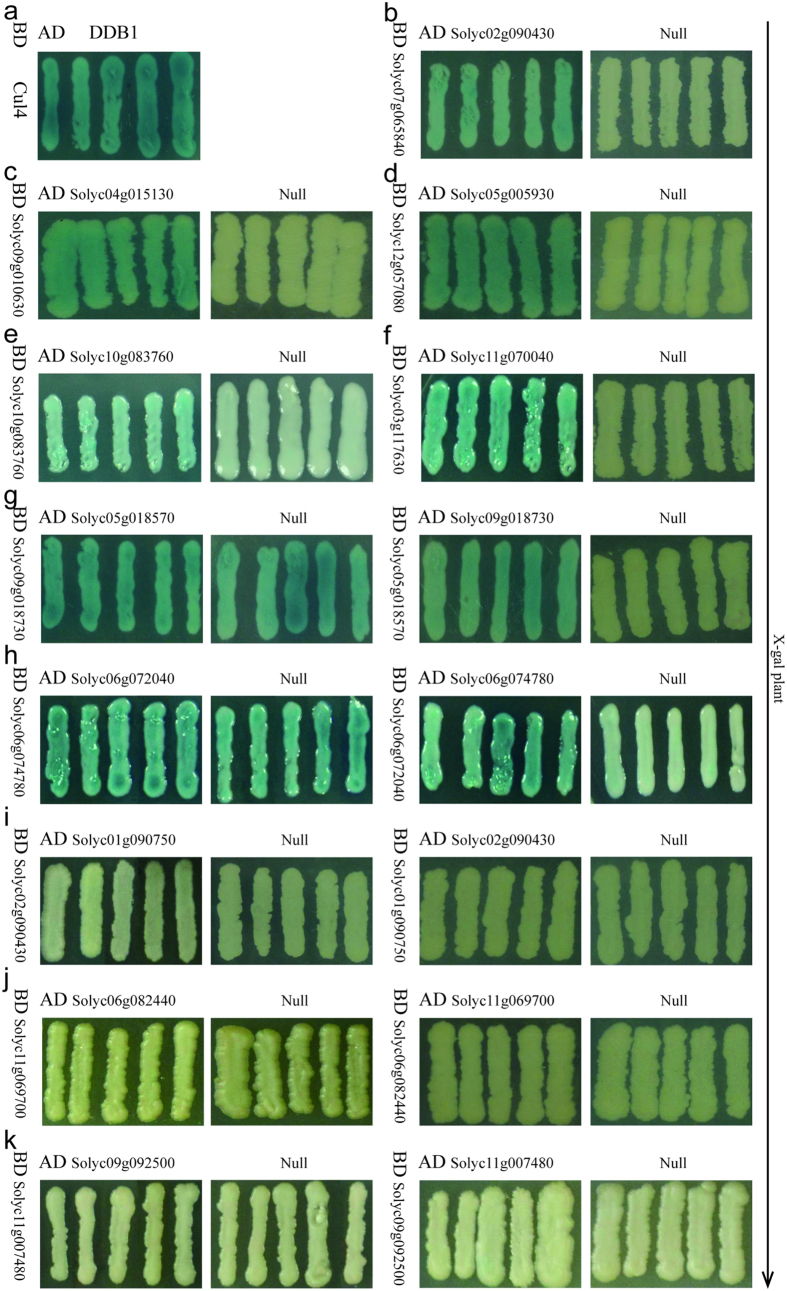
The interaction analysis from Y2H studies using the LacZ reporter. (**a**) The positive control, (**b–k**) The ten predicted PPIs. Self-activation occurs in groups (**g,h**), and no interactions occur in groups (**i–k**).

**Figure 6:**
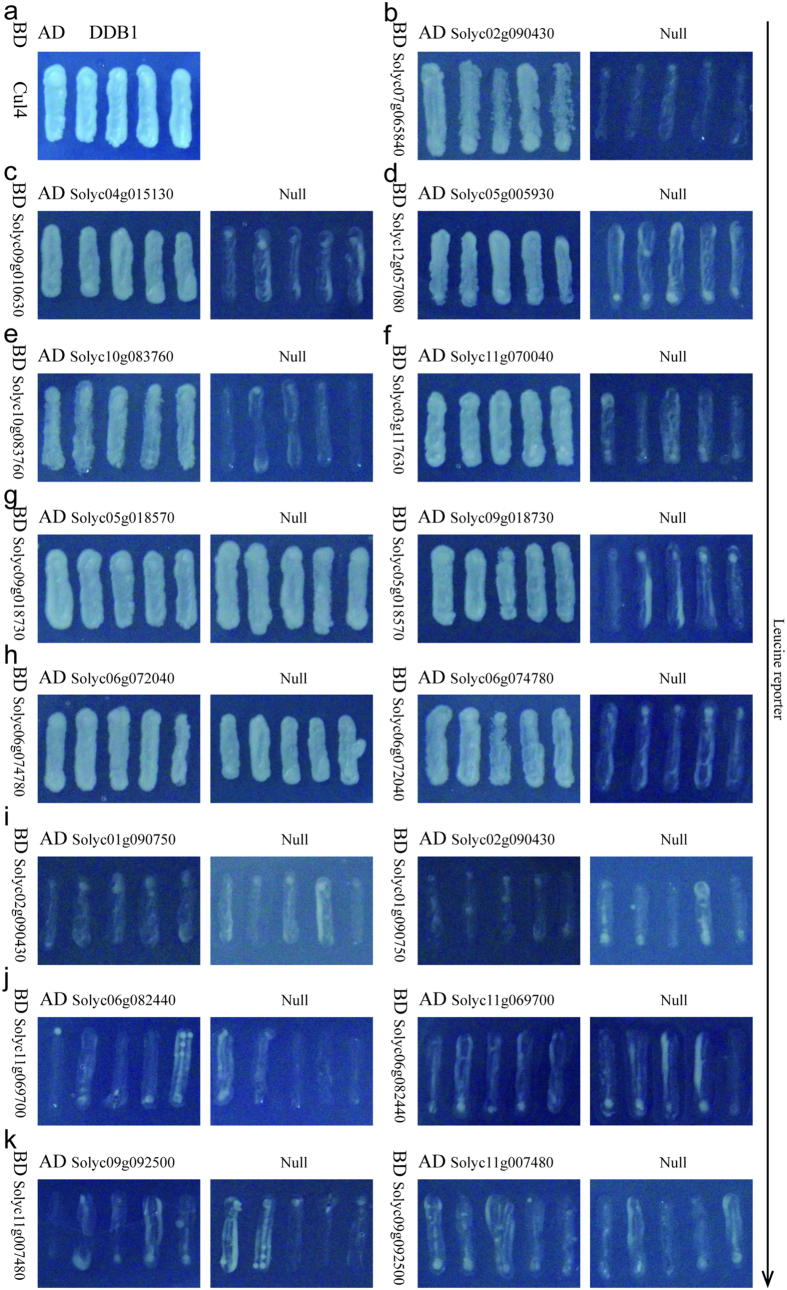
The interaction analysis from Y2H studies using the Leucine reporter. (**a**) The positive control, (**b–k**) The ten predicted PPIs. Self-activation occurs in groups (**g,h**), and no interactions occur in groups (**i–k**).

**Figure 7:**
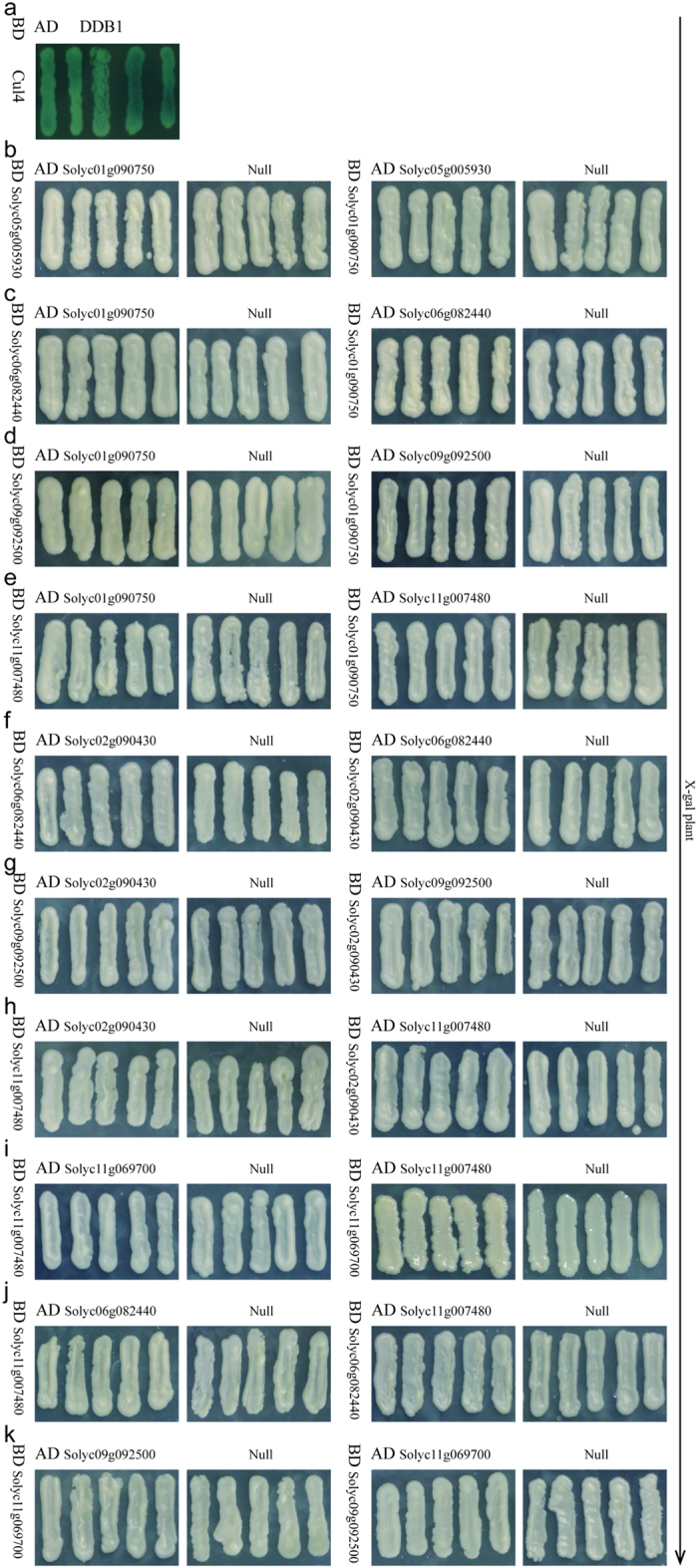
The interaction analysis from Y2H studies using the LacZ reporter. (**a**) The positive control, (**b–k**) The ten negative controls. No signals were detected.

**Figure 8:**
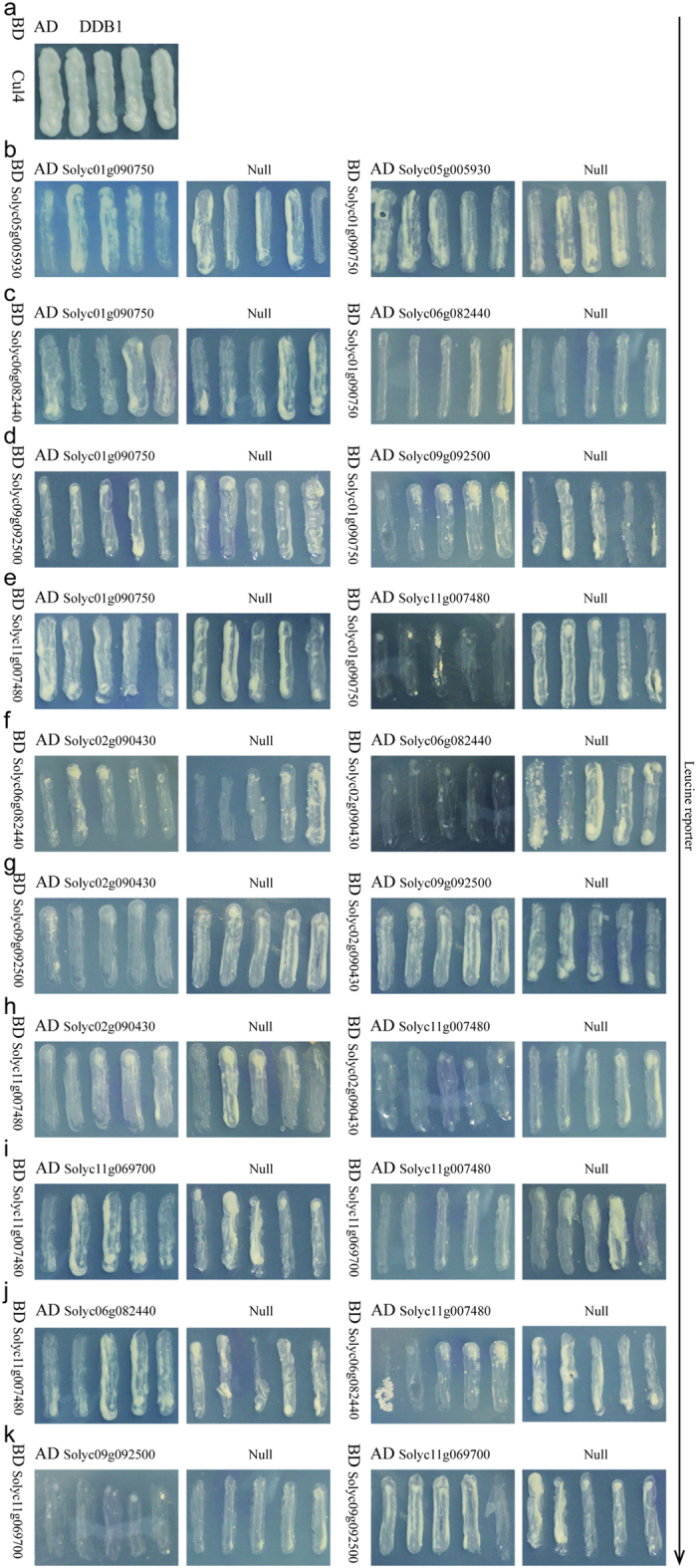
The interaction analysis from Y2H studies using the Leucine reporter. (**a**) The positive control, (**b–k**) The ten negative controls. No signals were detected.

**Figure 9:**
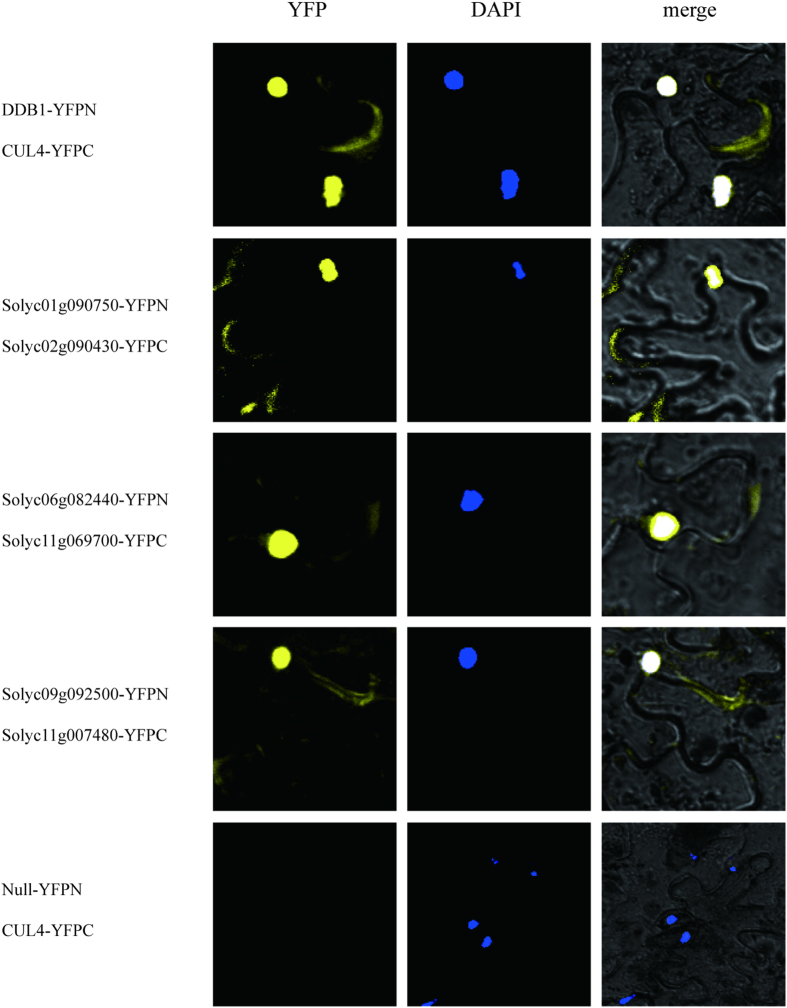
The interaction analysis using BiFC. Three interactions were visualized on the confocal microscopy images. Yellow indicates YFP fluorescence, and blue indicates nuclei stained with DAPI.

**Figure 10:**
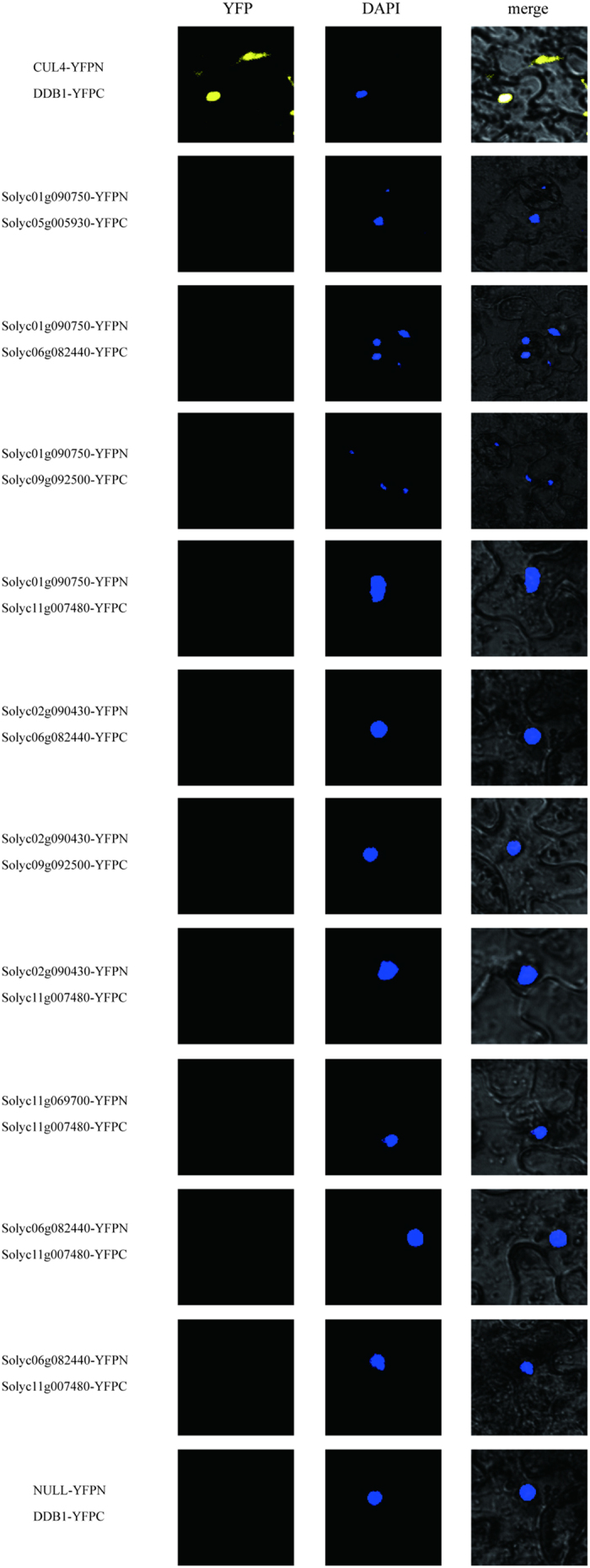
The interaction analysis using BiFC. No signals were detected.

Yeast (*Saccharomyces cerevisiae*) two-hybrid analysis^62^ complemented by biomolecular fluorescence complementation (BiFC) analysis^63^ was conducted to test the validity and accuracy of our predictions. Each pair of interacting proteins was used as bait and prey. Thirty-six protein interactions in the PTIR were randomly selected based on a single increment of 10000 from the first identified PPI (PTIR000001). Of these, a total of ten pairs (involving eighteen proteins) were used for the follow-up experiments with the consideration of their confidence values and the appropriateness of their protein length, which is convenient for the cloning of PCR products (Supplementary Table S5). The Y2H studies verified that seven pairs of proteins interacted without auto-transcriptional activation, including PTIR000001 (Solyc10g083760-Solyc10g083760), PTIR130001 (Solyc02g090430-Solyc07g065840), PTIR180001 (Solyc03g117630-Solyc11g070040), PTIR200001 (Solyc04g015130-Solyc09g010630), PTIR220001 (Solyc05g005930-Solyc12g057060), PTIR230001 (Solyc05g018570-Solyc09g018730), and PTIR270001 (Solyc06g072040-Solyc06g074780). Notably, the Y2H results from both the LacZ reporter (Fig. 5) and Leucine reporter (Fig. 6) were consistent. Additionally, ten pairs of proteins without predicted interactions were selected as negative controls, and no signal was detected (Figs 7 and 8). By contrast, no signal was detected from the remaining three predicted interacting partners, possibly because the interacting proteins could not be transported into the nucleus^64^. To overcome the limitation, we employed BiFC to investigate possible interactions of these three predicted PPIs in cells from tobacco (*Nicotiana Benthamiana*) plant leaves by transiently co-expressing the putatively interacted partners. BiFC has been widely applied to identify PPIs at the genome level via enhanced yellow fluorescent protein-based reconstruction^65^. In our studies, BiFC assays identified interacting signals from the three predicted PPIs [PTIR050001 (Solyc01g090750-Solyc02g090430), PTIR280001 (Solyc06g082440-Solyc11g069700), and PTIR330001 (Solyc09g092500-Solyc11g007480)] (Fig. 9). In addition, no signal was detected for the ten pairs of control proteins (Fig. 10). Collectively, these results confirm the genuine interactions between the selected protein pairs and suggest that our predictions have a very high accuracy rate.

### The PTIR scheme and interface

**Figure 11:**
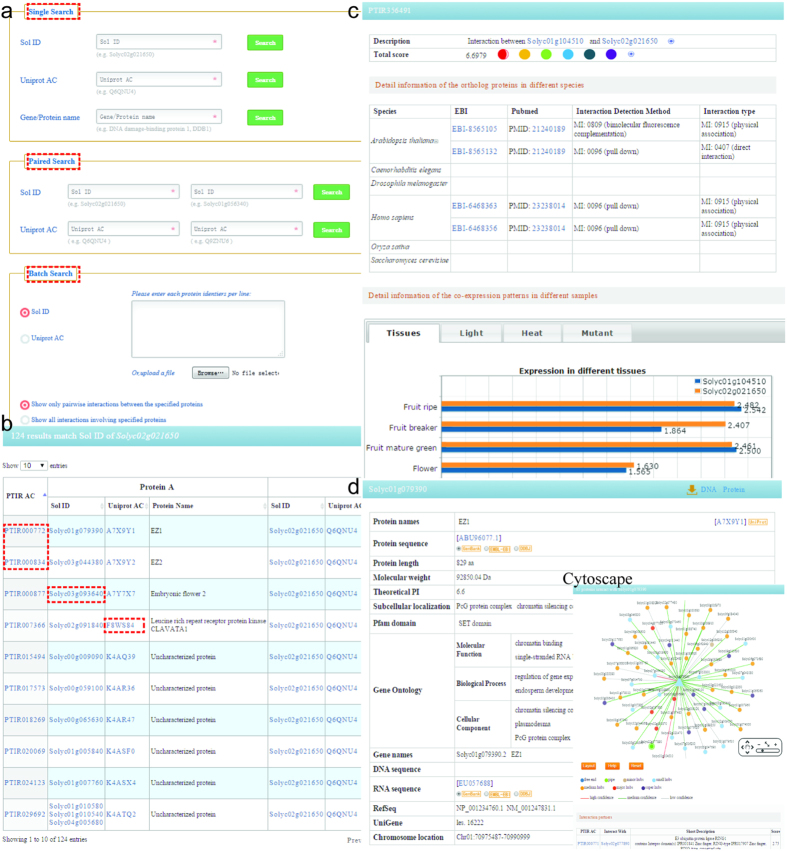
The interface of the PTIR. (**a**) Three search categories for querying. (**b**) The results are shown in a tabular format. Users can visualize the detailed information by clicking on the PTIR AC and/or Sol ID. (**c**) The PPI page. (**d**) The detailed information for a single protein.

For the convenience of using the predicted PPI data, we have developed a searchable database, Predicted Tomato Interactome Resource (PTIR). The general process of data identification, integration, annotation, statistics and database development is illustrated in the Methods section. Thereafter, users could find the PPIs of interest through simply entering a Sol ID or UniProt AC. Three search categories are provided: (1) Single Search; (2) Pair Search; and (3) Batch Search (http://bdg.hfut.edu.cn/ptir/search.html) (Fig. 11a).

The single search option provides an interface for querying the PTIR with accession numbers (Sol ID or UniProt AC) or the keywords of gene/protein names. The full name and abbreviation are both feasible, where they are automatically normalized with synonyms.

The pair search options are accession number centric. Users can find the potential PPIs with their identifiers of Sol ID and UniProt AC. Two identifiers in the text boxes should be consistent. Only if these two proteins interact with each other, the records will be found.

The batch search option allows users to input a list of protein identifiers (Sol ID or UniProt AC), or to upload a file containing accession numbers. Before submitting, the algorithms in the settings should be specified to show the interactions between the proteins involved or the interactions involving any one of the proteins.

After searching, the results are shown in a tabular format, containing PTIR AC, Sol ID (protein A), UniProt AC (protein A), Protein name (protein A), Sol ID (protein B), UniProt AC (protein B), Protein name (protein B) (Fig. 11b). From this table, users can browse the detailed information of PPIs and the interacting proteins involved by clicking on their corresponding links. In the PPI pages (Fig. 11c), the brief description of score, the information of interaction type and experimental methods for initial PPIs in the reference species are shown. Furthermore, Protein names, Protein sequence and length, Molecular weight (MW), Theoretical PI, Pfam domain, Gene names, DNA and RNA sequence, RefSeq, UniGene, Chromosome location, Subcellular localization and GO annotation are listed in the detailed description of each interacting protein (Fig. 11d). Access to the known databases’ interpretation of corresponding description is also provided. In addition, the Cytoscape Web^66^, a web browser applet, has been integrated into the web pages and used to visualize the interacting proteins (nodes) and potential interactions (edges), where the proteins are graphed as nodes (one dimension) and the interactions are graphed as edges (two dimension). It is hoped that these information will effectively aid users to explore the relationship between proteins of interest. Finally, users can interact with the network and save it in different formats (network maps or network data).

## Discussion

**Figure 12:**
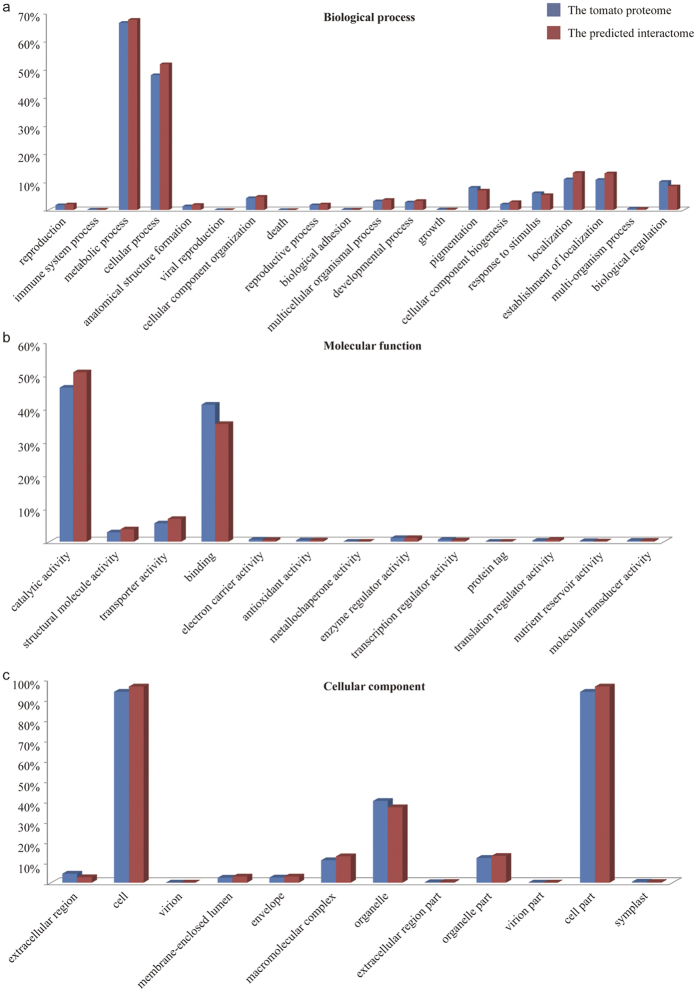
The protein categories of the interactome in comparison with the whole tomato genome using the GO Slim categories: (**a**) Biological Process, (**b**) Cellular Component, and (**c**) Molecular Function.

We predicted the tomato interactome from experimental protein interaction datasets of model organisms and revealed protein transactions and interacting complexes. As expected, many significant evolutionarily conserved pathways, such as DNA repair pathways and endomembrane trafficking systems, were found in tomatoes^67^,^68^. To verify whether the interacting proteins in our predicted interactome possess good coverage of the entire tomato proteome, we applied the GO annotation to compute and compare the protein distribution. The enrichments of GO terms for biological processes, cellular components, and molecular functions were statistically calculated (Fig. 12). We showed that the proteins in the PTIR and the proteins in the entire tomato proteome exhibit a consistent distribution pattern across the GO Slim categories. The Pearson’s correlation coefficients (*r*) for biological processes, molecular functions and cellular components between these two protein datasets were 0.9985 (*P*-value = 5.73 × 10^−28^; Fig. 12a), 0.9982 (P-value = 5.01 × 10^−16^; Fig. 12b), and 0.9992 (P-value = 2.47 × 10^−15^; Fig. 12c), respectively. These significant correlations strongly suggest that the proteins in the PTIR have good coverage of the entire proteome, further indicating that our predictions could facilitate understanding cellular networks.

Interestingly, in the GO-represented biological process, the proportions of some terms (cell growth and morphogenesis, metabolic process, and localization) in the PTIR were higher than those in the tomato proteome; conversely, the proportions of other terms (pigment and stress response) were lower than in the tomato proteome (Fig. 12a). These differences can be attributed to the plants’ unique features that evolved in response to photoautotrophic and environmental challenges. In addition, the PIN that underlies plant-specific functions was essentially missing because 90% of the interologs were based on animals. Nevertheless, we identified a variety of plant-specific complexes, such as light-harvesting and photosystem complexes (e.g., interaction between Solyc08g080050 and Solyc06g054260).

Animals far outnumber plants in the reference species. This imbalance not only reduced the number of predicted plant-specific complexes but also affected the final scores of the predicted PPIs. In our study, a complementary approach was applied to assess the reliability of each PPI based on computational methods, such as shared GO terms, co-evolution, co-expression, co-localization, and available DDIs. The comparison analysis of the construction method and assessment approach showed that they are totally independent events (*r* = 0.11, *P*-value < 0.01). By combining these approaches, we identified 12,291 high scores (greater than or equal to 7), 226,553 medium scores (between 2 and 7), and 119,102 low scores (less than 2). In the PTIR, there are four predicted interactions that overlap with the 16 experimentally reported interactions collected in the IntAct database. This proportion (25%) is in good agreement with the statistical value of 26.44% in PAIR^25^.

When the tomato genome sequence was completed in 2012, the annotation was performed using a pipeline based on PhyloFUN and Interpro2GO^69^. Notably, protein functions and attributes were previously assessed and compared according to GO catalogues. However, of the 34,727 protein-coding genes, approximately 22.4% were labeled “Unknown Protein”. In this case, the incomplete annotation could benefit from the analysis of protein functions but requires further expansion. To a certain extent, our predicted tomato interactome could also provide novel insights into protein functions because functionally similar proteins tend to cluster in biological networks. In our study, a guilt-by-association strategy, which has been employed successfully to study the interactomes of many species^70^,^71^, was used to predict protein functions by examining their interacting partners in the PTIR.

To evaluate the accuracy of the protein function predictions in the PTIR, a total of 7,695 proteins annotated by GO terms in the biological process category were used as testing data. Among them, 695 proteins were randomly selected as the targets with their annotations manually removed and the remaining 7,000 proteins were used as known proteins for prediction. Subsequently, the interacting partners of these target proteins were identified. Using the guilt-by-association strategy^70^,^71^, each target protein was assigned with a number of GO terms. As expected, many identical and/or similar GO terms were linked between the predicted functions and their original annotations (data provided in the website). According to the method described by Lin^72^, the semantic similarities of GO terms were measured and their average value was found to be 0.20, which is significantly higher than the value of 0.10 for a randomized connection (*P*-value < 1*E*-10, Wilcoxon test). This result confirms the usefulness of the PTIR for predicting protein function using the guilt-by-association strategy and the feasibility of predicting the functionality of unannotated proteins based on their interactions.

Therefore, this strategy was used to predict the most possible functional terms of proteins labeled “Unknown Protein” in the PTIR. The significantly enriched terms and their individual *P* values were detected using the hypergometric test in the GO Term Finder^73^. We reserved the top five predicted terms, which are sufficient to recover the known biological function of a given gene (data provided in the website)^74^. In total, we found that approximately 95.2% (2789/2931) of the “Unknown Proteins” in the PTIR could be annotated without recycling the annotation operation of our prediction.

**Figure 13:**
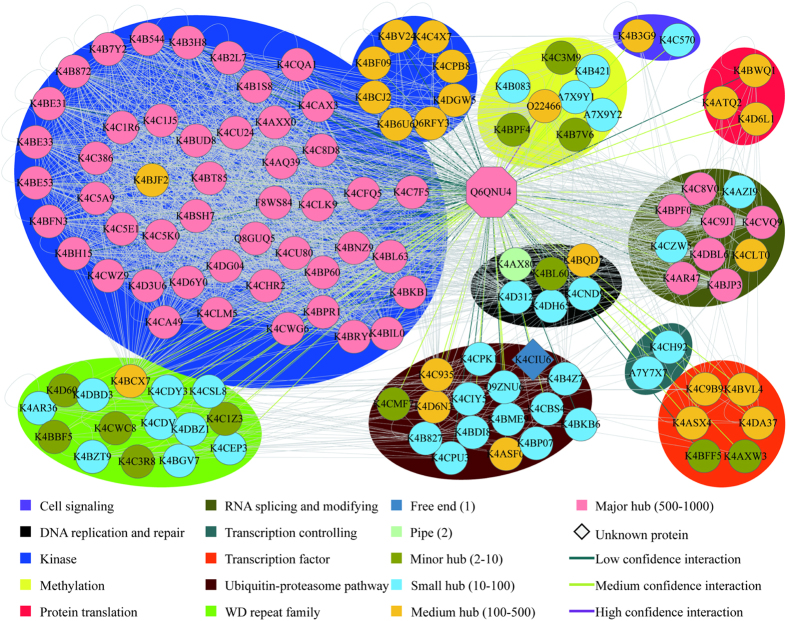
The PPIs related to DDB1, UniProt AC: Q6QNU4.

A case study of DNA damage-binding protein 1 (DDB1, UniProt AC: Q6QNU4, Sol ID: Solyc09g031610.2.1), revealed 124 interacting partners (Supplementary Table S6). Most fall into known complexes, such as Ubiquitin-proteasome pathway/DNA repair (e.g., DET1 and CUL4), WD repeat family (e.g., COP1), and RNA splicing and modifying (e.g., AGO1 and CDC5; Fig. 13)^75^,^76^,^77^,^78^. A protein without a previously annotated function (UniProt AC: K4CIU6, Sol ID: Solyc08g008120.2.1) had an interaction with DDB1. Based on the hypothesis that interacting proteins tend to be involved in the same pathway, the function of K4CIU6 was inferred according to its interaction with DDB1, which possesses six distinct biological process annotations, including red, far-red light phototransduction (GO: 0009585), embryo development ending in seed dormancy (GO: 0009793), negative regulation of transcription, DNA-templated (GO: 0045892), protein ubiquitination (GO: 0016567), negative regulation of photomorphogenesis (GO: 0010100), and red or far-red light signaling pathway (GO: 0010017). As a result, the five possible functions of K4CIU6 are listed in Table 4. Consistently, the predicted function of ubiquitination was supported by the fact that its Arabidopsis ortholog (UniProt AC: Q9FFS4, TAIR ID: At5g41560) is annotated as positive regulation of proteasomal ubiquitin-dependent protein catabolic process (GO: 0032436)^54^. Therefore, this type of annotation can be used to assign putative members based on their interacting partners and deduce molecular functions of the unknown proteins in the tomato genome.

Another case study of protein function prediction is also proved to be successful by integrating information from existing studies. A previously reported FR database^79^ collected hundreds of experimentally verified proteins by manual curation from the literature and eleven of those proteins, without annotation in the biological process category, were found in the PTIR. As shown in Table 5, eight proteins have updated annotations in the UniProt database since the release of the FR database 1.0. Interestingly, the predicted function of most of the proteins was supported by the literature and/or the updated annotation. For example, Fw2.2 (UniProt AC: Q9LKV7) participates in a cell-cycle control signal transduction pathway^80^ and was predicted to be involved in G2/M transition of mitotic cell cycle (GO: 0010971). This prediction may provide clues to its biological function. Similarly, NDPS1 (UniProt AC: C1K5M2) is involved in the synthesis of long-chain polyisoprenoids according to biochemical experiments^81^ and its annotation was updated with the metabolic process (GO: 0008152) category in the UniPort database. Comparatively, the description of pentacyclic triterpenoid biosynthetic process (GO: 0019745) in the PTIR is more useful because it provides specific functional information than its father term (GO: 0008152).

In summary, the PTIR was constructed and implemented as an easy-to-use affordable Web-based tool for the analysis of the tomato PIN, based on the evolutionary conservation of interacting proteins and their interactions across species. Each identified PPI was assigned a confidence score according to the total value of the sequence information and biological function. This increases the validity and reliability of the interactome. Although the PTIR still has limited coverage of the tomato interactome, it is sufficient to provide comprehensive information on the highly conserved protein networks and shed light on the functions of protein interactions. Collectively, these protein interactions could be used by both theorists and experimentalists to reassemble protein complexes, expand existing pathways and enrich genome annotation, thereby improving the understanding of biological processes at the systems level.

## Methods

### Data sets

The interactome datasets of Arabidopsis (18,462 pairs), nematode worm (20,472 pairs), fruit fly (30,578 pairs), human (151,226 pairs), rice (699 pairs) and yeast (126,097 pairs) were downloaded from the IntAct database (06-15-2014 release; http://www.ebi.ac.uk/intact/)^15^. These PPIs have been experimentally determined. Orthologous clusters were detected by the ortholog predicting algorithm, INPARANOID (version 8, bit score cutoff = 40 bits and sequence overlap cutoff = 0.5; http://inparanoid.sbc.su.se/cgi-bin/index.cgi)^82^,^83^. The numbers of orthologs between the tomato and reference species (Arabidopsis, nematode worm, fruit fly, human, rice and yeast) were 24,670, 19,683, 16,689, 23,943, 33,648, and 7,837, respectively.

### Plant materials

The tomatoes (*Solanum lycopersicum* cv. Ailsa Craig, LA2838A) were obtained from Tomato Genetics Resource Center (Davis, CA). The tomato plants were germinated and grown in a greenhouse under artificial conditions (26 °C day, 18 °C night; 16 hours light, 8 hours dark). The harvested tomato tissues, including the young leaves, flowers and fruits at various developmental stages, were immediately frozen in liquid nitrogen and stored at −80 °C prior to nucleic acid isolation and gene cloning.

### Flow chart of the PTIR

**Figure 14:**
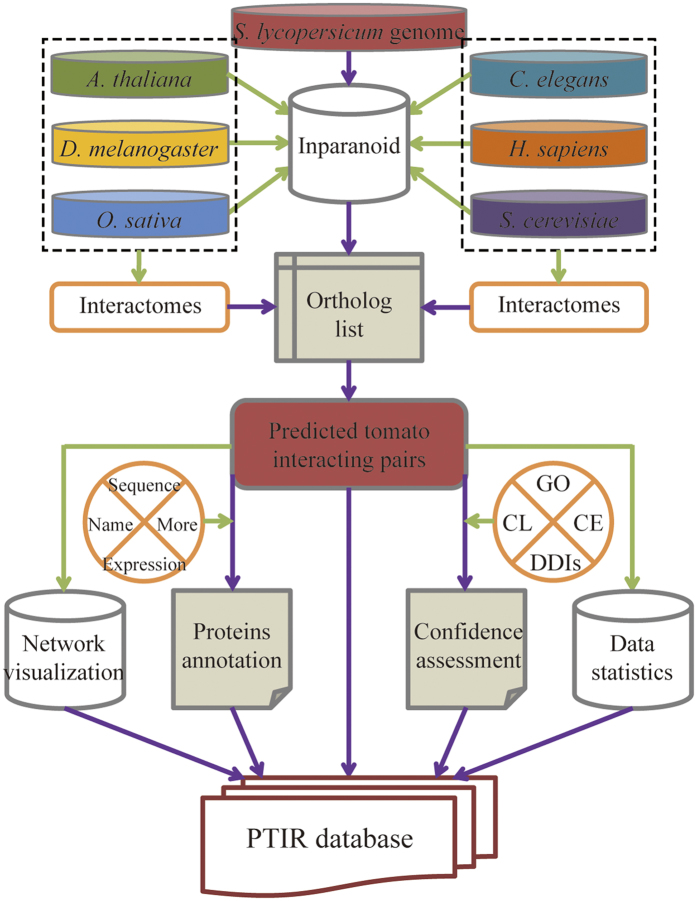
The PTIR scheme. GO: Gene Ontology; DDIs: Domain-Domain Interactions; CL: Cellular Localization; CE: Co-expression.

The orthologs were mapped onto interactome datasets of the reference species and locations where any two tomato proteins mapped with reference species were recorded as interacting protein groups. The UniProt AC was used as cross-identification between the interactome and ortholog datasets. After mapping, the confidence of each PPI was evaluated. The proteins involved were mainly annotated in two parts: the protein annotation [e.g., name/synonyms, nucleotide sequences, protein sequences, CDS site, theoretical PI and MW (molecular weight), PIRSF, Pfam, SUPFAM, and Prosite annotation] and the interaction map (e.g,. the co-expression score value, the experiment in reference species where the tomato PPI was predicted from). The general process is outlined in Fig. 14. Finally, the PTIR was implemented in PHP + MySQL + JavaScript and is freely available.

### Study of subcellular localization

Protein subcellular localization data were first obtained from the UniProt database^57^ available in the “Subcellular location” section (http://www.uniprot.org/). Only the entries with labels in the following evidence codes were reserved^43^: EXP (Inferred from Experiment), IDA (Inferred from Direct Assay), IEP (Inferred from Expression Pattern), IMP (Inferred from Mutant Phenotype), IC (Inferred by Curator), IEA (Inferred from Electronic Annotation), RCA (Inferred from Reviewed Computational Analysis), and ISS (Inferred from Sequence or Structural Similarity). Otherwise, protein subcellular localization was assigned based on the predicted presence of any N-terminal presequences through TargetP software using default parameter values^58^. Based on these data, proteins were localized to the following 13 distinct subcellular compartments: apoplast, cell wall, chloroplast, cytoplasm, cytoskeleton, endoplasmic reticulum (ER), Golgi, membrane, mitochondria, nucleus, ribosome, secreted, and vacuole. Some proteins located in several clear compartments are also listed. However, if the location was not clear, proteins were assigned as “undefined”, and proteins with no localization information were assigned as “unknown”. Finally, the protein subcellular localization information from all the sources was integrated together. Considering that two interacting proteins may be located in physically adjacent compartments (i.e., cytosol-membrane associated) or show trafficking interactions (i.e., nucleus-cytosol), we uniformly assigned these adjacent sites as one group according to the records (e.g., Golgi apparatus/ER group, Golgi apparatus/vacuole group)^23^. If two interacting proteins share any one of the locations within a group, the PPI will score a point.

### Analysis of protein co-expression

The transcriptome datasets of the tomato gene transcription profiles were downloaded from the GEO repository and Sequence Read Archive (SRA) database. They were generated using high-throughput technologies, such as microarrays and RNA-seq, and were derived from various samples covering different tissues, developmental stages, stress treatments and mutants. In total, 96 samples were gathered and calculated according to their expression levels. Because not all the proteins harbored in our predicted tomato interactome were found in every sample, we separately graded each PPI of each sample:


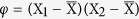


Here, X_1_ and X_2_ represent the two values of each member in an interacting protein pair, where 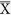 is the mean value of each sample. Then, the Pearson Correlation Coefficient score (*γ*) was assigned according to the value (*φ*):


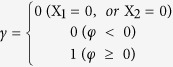


where 1 indicates that the gene expression patterns of the two interacting proteins are consistent, and 0 means that they are inconsistent. The final CS for the confidence of the PPIs in our predicted interactome was the average 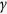 value from each sample:


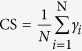


where *N* is the number of expression samples (96 here). The values of CS range from 0 to 1, and a high value indicates a high-confidence protein interaction, and a low value indicates a low-confidence protein interaction.

### Y2H assays

The Y2H assays were performed using the MATCHMAKER GAL4 Two-Hybrid System III according to a procedure described in *Current Protocols in Protein Science*^84^. The open reading frames of eighteen target genes were amplified by PCR with each primer pair carrying various restriction enzyme sites (Supplementary Table S7). The PCR products were digested and cloned into pEG202 and/or pJG4-5 to create bait and/or prey construct vectors. Then, the different combinations of bait and prey constructs were co-transfected into the yeast strain EGY48. The cells were plated on yeast medium lacking histidine (-H), tryptophan (-W), uracil (-U) and leucine (-L). After 2 to 4 days, these yeast strains were tested on selective plate medium to analyze the presence of interactions. The plates were incubated for 3 days at 28 °C to cause the yeast to turn blue on medium containing 40 μg/ml X-gal. Empty prey and bait vectors were used as a negative control and positive controls (DDB1 and Cul4) were also cultured^85^. The assays were repeated at least twice to increase the experimental credibility and decrease error.

### BiFC analysis

The coding sequences of six target genes were amplified with gene-specific primer sets harboring multiple restriction sites (Supplementary Table S8). The PCR products were cloned into 35S-pBI-NBi or 35S-pBI-CBi plasmids to construct vectors. These constructs containing the cDNA with the fusion proteins were injected into cells from tobacco (*N. benthamiana*) plant leaves by *Agrobacterium*-mediated infiltration^86^. After at least 48 hours, the epidermal cell layers were fixed and counter-stained with 4′,6-diamidino-2-phenylindole (DAPI). Subsequently, the cells were visualized on an Olympus FV1000 microscope with excitation = 488 nm and emission = 500/100 nm. In parallel, 35S-pBI-Cul4-NBi and 35S-pBI-CBi were used as a negative control, and 35S-pBI-Cul4-NBi and 35S-pBI-DDB1-CBi were included as a positive control^85^.

## Additional Information

**How to cite this article**: Yue, J. *et al.* PTIR: Predicted Tomato Interactome Resource. *Sci. Rep.* **6**, 25047; doi: 10.1038/srep25047 (2016).

## Supplementary Material

Supplementary Tables

## Figures and Tables

**Table 1:** The PPIs of the different species gathered from public databases.

Organisms	IntAct	BioGRID	DIP	STRING
human	154,230	186,469	7,399	926,131
yeast	126,303	230,197	24,574	217,656
worm	20,481	8,076	4,125	317,530
fruit fly	45,662	39,308	23,261	419,282
Arabidopsis	18,763	17,780	446	560,881
rice	707	3	0	1,431,236
wheat	82	0	0	0
maize	49	1	0	0
tomato	148	0	0	0

**Table 2:** The average interacting partners generated from the different databases.

Species	No. of Proteins	No. of Interactions	Average	Release Year	Reference
*Solanum lycopersicum*	10,626	357,946	34	–	–
*Arabidopsis thaliana*	3,617	19,979	6	2007	Giesler-Lee *et al.*^23^
*Arabidopsis thaliana*	10,380	149,900	14	2011	Lin *et al.*^25^
*Arabidopsis thaliana*	11,931	201,699	17	2012	Wang *et al.*^24^
*Brassica rapa*	20,677	740,565	36	2013	Yang *et al.*^27^
*Oryza sativa*	5,049	76,585	15	2011	Gu *et al.*^26^
*Zea mays*	14,000	2,762,560	197	2016	Zhu *et al.*^28^

**Table 3:** A series of high-quality datasets and the frequency and enrichment of high-confidence interactions.

MI-score	Dataset	Number of PPIs	High confidence PPIs	Frequency	*P* value
0.9	High_quality_0.9	1,585	882	0.5565	0
0.8	High_quality_0.8	2,976	1,150	0.3864	0
0.7	High_quality_0.7	6,839	1,584	0.2316	0
0.6	High_quality_0.6	16,957	3,004	0.1772	0
0.5	High_quality_0.5	50,020	4,983	0.0996	0
0.4	High_quality_0.4	115,406	7,709	0.0670	0
0.3	High_quality_0.3	348,132	12,029	0.0346	8.0E-06
0.2	High_quality_0.2	357,895	12,288	0.0343	0.963
0.1	High_quality_0.1	357,932	12,291	0.0343	1

The *P* value is reported by hypergeometric test.

**Table 4:** The predicted function of protein Solyc08g008120 (UniProt AC: K4CIU6).

Predicted function in our study	*P* value
GO:0010017: red or far-red light signaling pathway	0.001
GO:0010100: negative regulation of photomorphogenesis	0.003
GO:0045892: negative regulation of transcription, DNA-templated	0.015
GO:0009793: embryo development ending in seed dormancy	0.017
GO:0016567: protein ubiquitination	0.043

**Table 5:** The predicted functions of 11 proteins compared with their descriptions from the literature and updated annotations.

UniProt	The most related prediction	The recently updated annotation	Functional description from FR
C1K5M2	GO:0019745:pentacyclic triterpenoid biosynthetic process	GO:0008152:metabolic process	It is involved in the synthesis of long-chain polyisoprenoids.
C8C507	GO:0009734:auxin-activated signaling pathway	No annotation	TIR1 and its homologues act as auxin receptors and play a crucial role in auxin-mediated plant development.
H9D2D6	GO:0007165:signal transduction	GO:0052544:defense response by callose deposition in cell wall	It is involved in AsA biosynthesis to regulate ascorbic acid concentration.
K4C9J1	GO:0006950:response to stress GO:0042254:ribosome biogenesis	No annotation	It plays important roles in tomato development and virus defense by participating in RNA induced silencing complex.
K4CA50	GO:0019745:pentacyclic triterpenoid biosynthetic process	GO:0008152:metabolic process	It is involved in the synthesis of long-chain polyisoprenoids.
K4D3U9	GO:0019745:pentacyclic triterpenoid biosynthetic process	GO:0008152:metabolic process	It is involved in the synthesis of long-chain polyisoprenoids.
Q5UNS1	GO:0006950:response to stress	GO:0008152:metabolic process	It plays an important role in the chilling resistance process.
Q5UNS2	GO:0006950:response to stress	GO:0008152:metabolic process	It plays an important role in the chilling resistance process.
Q9LKV7	GO:0010971:positive regulation of G2/M transition of mitotic cell cycle	No annotation	It participates in a cell-cycle control signal transduction pathway and negatively regulates fruit size by interacting with LeCK II β1.
Q9S7H9	GO:0009735:response to cytokinin	GO:0042127:regulation of cell proliferation	It is involved in transducing the signals leading to fruit growth by cell divisions.
Q9SMD5	GO:0000082:G1/S transition of mitotic cell cycle GO:0007050:cell cycle arrest	GO:0000082:G1/S transition of mitotic cell cycle GO:0042127:regulation of cell proliferation	It is involved in transducing the signals leading to fruit growth by cell divisions.
