# Supplementary material for: PTIR: Predicted Tomato Interactome Resource
Source: Sci Rep. 2016 Apr 28;6:25047. doi: 10.1038/srep25047 (PMC4848565; doi:10.1038/srep25047)
Supplement: Supplementary Tables [file srep25047-s1.pdf]

Supplementary Tables

PTIR: Predicted Tomato Interactome Resource

Junyang Yue[1,†], Wei Xu[1,†], Rongjun Ban[2,†], Shengxiong Huang[1], Min Miao[1], Xiaofeng Tang[1], Guoqing Liu[1], and Yongsheng Liu[1,3,*]

[1]School of Biotechnology and Food Engineering, Hefei University of Technology, Hefei 230009, China

[2]School of Information Science and Technology, University of Science and Technology of China, Hefei 230026, China

[3]Ministry of Education Key Laboratory for Bio-resource and Eco-environment, College of Life Science, State Key Laboratory of Hydraulics and Mountain River Engineering, Sichuan University, Chengdu 610064, China

[†] These authors contributed equally to this work.

[*] Corresponding author: Tel: +86 18709832886; E-mail: liuyongsheng1122@hfut.edu.cn

Table S1. The most (more than 1000) highly connected protein interaction hubs.

Sol ID	Uniprot AC	Protein Name	Description	Connections
Solyc09g010630	K4CR90	Uncharacterized protein	Heat shock protein	3,751
Solyc10g086410	K4D473	Uncharacterized protein	Heat shock protein 70-3	3,751
Solyc11g066060	K4D9L5	Uncharacterized protein	Heat shock protein	3,751
Solyc10g086410	P27322	Heat shock cognate 70 kDa protein 2	Heat shock protein 70-3	3,751
Solyc04g011440	H1ZXA9	Heat shock protein 70 isoform 3	Heat shock protein	3,748
Solyc03g117630	K4BLR5	Uncharacterized protein	Heat shock protein	3,748
Solyc06g076020	K4C9W3	Uncharacterized protein	Heat shock protein	3,748
Solyc07g005820	K4CB53	Uncharacterized protein	Heat shock protein	3,748
Solyc11g066100	K4D9L9	Uncharacterized protein	Heat shock protein	3,748
Solyc06g076020	P24629	Heat shock cognate 70 kDa protein 1	Heat shock protein	3,748
Solyc03g007890	K4BEL1	Uncharacterized protein	Heat shock protein 90	2,415
Solyc06g036290	K4C4Z6	Uncharacterized protein	Heat shock protein 90	2,414
Solyc07g065840	P36181	Heat shock cognate protein 80	Heat shock protein 90	2,414
Solyc12g015880	Q6UJX4	Molecular chaperone Hsp90-1	Heat shock protein 90	2,414
Solyc02g080470	K4B9R8	Uncharacterized protein	Heat shock protein 4	2,368
Solyc12g043110	K4DF99	Uncharacterized protein	Heat shock protein 4	2,368
Solyc12g043120	K4DFA0	Uncharacterized protein	Heat shock protein 4	2,368
Solyc11g069700	K4DAC6	Elongation factor 1-alpha	Elongation factor 1-alpha	1,437
Solyc06g005060	P17786	Uncharacterized protein	Elongation factor 1-alpha	1,437
Solyc06g009960	K4C424	Uncharacterized protein	Elongation factor 1-alpha	1,435
Solyc04g009770	K4BPD2	Uncharacterized protein	DNAJ chaperone	1,179
Solyc05g055160	K4C2J3	Uncharacterized protein	DNAJ chaperone	1,179
Solyc11g006460	K4D4Q9	Uncharacterized protein	DNAJ chaperone	1,179
Solyc01g090550	K4AYZ6	Uncharacterized protein	DNAJ chaperone	1,176
Solyc11g071830	K4DAP0	Uncharacterized protein	DNAJ chaperone	1,176
Solyc01g106210	K4B2I9	Uncharacterized protein	Chaperone protein dnaj	1,031
Solyc01g106260	K4B2J4	Uncharacterized protein	Chaperone DnaK	1,031

Table S2. The enrichment (Hypergometric test) and frequency of each GO term annotation (molecular function) for large hubs and free ends were calculated.

GO Term	Description	Large Hubs		Free Ends	
		P Value	Frequency	*P* Value	Frequency
GO:0003824	catalytic activity	0.9993094	0.3280632	0.738	0.4121916
GO:0005198	structural molecule activity	2.01E-06	0.0869565	1	0.0101597
GO:0005215	transporter activity	0.9927828	0.0237154	0.8820479	0.0435414
GO:0005488	binding	0.0002233	0.5375494	0.33629	0.4354136
GO:0009055	electron carrier activity	1	0	2.42E-07	0.0304790
GO:0015457	auxiliary transport protein activity	1	0	1	0
GO:0016209	antioxidant activity	1	0	1.18E-05	0.0203193
GO:0016530	metallochaperone activity	1	0	1	0
GO:0030234	enzyme regulator activity	1	0	0.780203	0.0072569
GO:0030528	transcription regulator activity	0.9994851	0.0039526	0.2783755	0.0333817
GO:0031386	protein tag	1	0	1	0
GO:0045182	translation regulator activity	0.5654463	0.0079051	1	0
GO:0045735	nutrient reservoir activity	1	0	1	0
GO:0060089	molecular transducer activity	0.3795255	0.0118578	0.7254857	0.0072569

Table S3. The enrichment (Hypergometric test) and frequency of each GO term annotation (biological process) for large hubs and free ends were calculated.

GO Term	Description	Large Hubs		Free Ends	
		P Value	Frequency	*P* Value	Frequency
GO:0000003	reproduction	0.0776388	0.0244898	0.7887858	0.0132656
GO:0022414	reproductive process	0.0707422	0.0244898	0.879861	0.0120025
GO:0001906	cell killing	1	0	1	0
GO:0002376	immune system process	0.209082	0.0061224	0.4004306	0.0037903
GO:0008152	metabolic process	0.7421576	0.2489796	0.8975242	0.2476311
GO:0009987	cellular process	0.3786618	0.2326531	0.907691	0.2128869
GO:0010926	anatomical structure formation	0.0002890	0.0326531	0.968156	0.0075805
GO:0016032	viral reproduction	0.115264	0.0020408	1	0
GO:0016043	cellular component organization	0.3080503	0.0387755	0.2886416	0.0366393
GO:0016265	death	0.0900918	0.0040816	0.8151419	0.0006317
GO:0022610	biological adhesion	1	0	0.4482554	0.0012634
GO:0032501	multicellular organismal process	0.4689761	0.0306122	0.3108712	0.0315856
GO:0032502	developmental process	0.3255522	0.0306122	0.1505548	0.0309539
GO:0040007	growth	0.0863818	0.0102041	0.2255958	0.0063171
GO:0040011	locomotion	1	0	1	0
GO:0043473	pigmentation	0.9995817	0.0285714	0.0015382	0.0777006
GO:0044085	cellular component biogenesis	0.0010112	0.0367347	0.1912033	0.0189514
GO:0048511	rhythmic process	1	0	0.3765615	0.0006317
GO:0050896	response to stimulus	0.0198860	0.0612245	0.0176836	0.0518004
GO:0051179	localization	0.9638745	0.0387755	0.9503754	0.0467467
GO:0051234	establishment of localization	0.9570261	0.0387755	0.9470427	0.0461150
GO:0051704	multi-organism process	0.1076812	0.0142857	0.2225509	0.0101074
GO:0065007	biological regulation	0.9995901	0.0326531	0.0008682	0.0852811

Table S4. The high-throughput datasets used in the process of building the PTIR.

Series Accession	Sample Number	Title	Platform	Authors
GSE33507	9	Transcriptome sequencing of 7 tissues of S. lycopersicum and 2 of its closely related wild ancestor S. pimpinellifolium	454 GS FLX Titanium	Pietrella *et al*.
GSE40257	3	Identification of direct target genes of the tomato fruit-ripening regulator RIN by promoter ChIP-chip	NimbleGen Tomato ITAG2 gene promoters 720K array	Fujisawa *et al*.
GSE40854	4	Synchronization of flowering transition by the tomato TERMINATING FLOWER gene	Illumina Genome Analyzer Iix	MacAlister *et al*.
GSE45774	52	Transcriptome Sequencing in wild and domesticated tomato species	Illumina Genome Analyzer II	Koenig *et al*.
GSE49125	6	Identification of direct target genes of the tomato FRUITFULL homologs during ripening by promoter ChIP-chip	NimbleGen Tomato ITAG2 gene promoters 720K array	Fujisawa *et al*.
GSE49289	18	Transcriptome profiling of tomato fruits of a FRUITFULL-suppressed line and rin mutant by next generation RNA sequencing	Illumina HiSeq 2000	Fujisawa *et al*.
GSE56610	2	Expression of orthologs of ribosome biogenesis factors in vegetative and reproductive tissues in Solanum lycopersicum	Illumina HiSeq 2000	Simm *et al*.
GSE56611	2	The composition and function of chaperone networks in Solanum lycopersicum explored by transcriptome sequencing and microarray meta-analysis	Illumina HiSeq 2000	Simm *et al*.

Table S5. Ten selected pairs of the predicted interacting proteins for the follow-up experiments.

PTIR AC	Sol ID(A)	Sol ID(B)	Protein Length(A)	Protein Length(B)	Score
PTIR000001	Solyc10g083760	Solyc10g083760	606	606	9.9063
PTIR050001	Solyc01g090750	Solyc02g090430	545	638	5.2188
PTIR130001	Solyc02g090430	Solyc07g065840	638	699	5.5313
PTIR180001	Solyc03g117630	Solyc11g070040	654	478	3.2708
PTIR200001	Solyc04g015130	Solyc09g010630	607	649	6.8333
PTIR220001	Solyc05g005930	Solyc12g057060	446	486	6.25
PTIR230001	Solyc05g018570	Solyc09g018730	417	315	5.7188
PTIR270001	Solyc06g072040	Solyc06g074780	232	143	6.7813
PTIR280001	Solyc06g082440	Solyc11g069700	419	447	5.4063
PTIR330001	Solyc09g092500	Solyc11g007480	470	453	5.75

Table S6. The interacting partners of protein DDB1.

Uniprot AC	Protein Name	Gene Name	Sol ID
A7X9Y1	EZ1	Solyc01g079390.2;EZ1	Solyc01g079390
A7X9Y2	EZ2	EZ2;Solyc03g044380.2	Solyc03g044380
A7Y7X7	Embryonic flower 2	LOC100134887;Solyc03g093640.2	Solyc03g093640
F8WS84	Leucine rich repeat receptor protein kinase CLAVATA1	SlmCLV1;Solyc02g091840.2;SlpCLV1	Solyc02g091840
K4AQ39	Uncharacterized protein	Solyc00g009090.2	Solyc00g009090
K4AR36	Uncharacterized protein	Solyc00g059100.2	Solyc00g059100
K4AR47	Uncharacterized protein	Solyc00g065630.1	Solyc00g065630
K4ASF0	Uncharacterized protein	Solyc01g005840.2	Solyc01g005840
K4ASX4	Uncharacterized protein	Solyc01g007760.2	Solyc01g007760
K4ATQ2	Uncharacterized protein	Solyc01g010580.2;Solyc01g010540.2;Solyc04g005680.2	Solyc01g010580; Solyc01g010540;
K4AX80	Uncharacterized protein	Solyc01g068340.2	Solyc01g068340
K4AXW3	Uncharacterized protein	Solyc01g080690.2	Solyc01g080690
K4AXX0	Uncharacterized protein	Solyc01g080770.2	Solyc01g080770
K4AZI9	Uncharacterized protein	Solyc01g094480.2	Solyc01g094480
K4B083	Uncharacterized protein	Solyc01g096950.2	Solyc01g096950
K4B1S8	Uncharacterized protein	Solyc01g103530.2	Solyc01g103530
K4B2L7	Uncharacterized protein	Solyc01g106500.2	Solyc01g106500
K4B3G9	Uncharacterized protein	Solyc01g109560.2	Solyc01g109560
K4B3H8	Uncharacterized protein	Solyc01g109650.2	Solyc01g109650
K4B421	Uncharacterized protein	Solyc01g111590.2	Solyc01g111590
K4B4Z7	Uncharacterized protein	Solyc02g021470.2;cul4	Solyc02g021470
K4B544	Uncharacterized protein	Solyc02g023950.2	Solyc02g023950
K4B6U6	Uncharacterized protein	Solyc02g065110.2	Solyc02g065110
K4B7V6	Uncharacterized protein	Solyc02g069740.2	Solyc02g069740
K4B7Y2	Uncharacterized protein	Solyc02g070000.2	Solyc02g070000
K4B827	Uncharacterized protein	Solyc02g070460.2	Solyc02g070460
K4B872	Uncharacterized protein	Solyc02g070910.1	Solyc02g070910
K4BBF5	Uncharacterized protein	Solyc02g086470.2	Solyc02g086470
K4BCJ2	Uncharacterized protein	Solyc02g090430.2	Solyc02g090430
K4BCX7	Uncharacterized protein	Solyc02g091790.2	Solyc02g091790
K4BDI8	Uncharacterized protein	Solyc02g093920.2	Solyc02g093920
K4BE31	Uncharacterized protein	Solyc03g006080.2	Solyc03g006080
K4BE33	Uncharacterized protein	Solyc03g006100.2	Solyc03g006100
K4BE53	Uncharacterized protein	Solyc03g006300.1	Solyc03g006300
K4BF09	Uncharacterized protein	Solyc03g025360.2	Solyc03g025360
K4BFF5	Uncharacterized protein	Solyc03g031830.1	Solyc03g031830
K4BFN3	Uncharacterized protein	Solyc03g033610.1	Solyc03g033610
K4BGV7	Uncharacterized protein	Solyc03g059100.1	Solyc03g059100
K4BH15	Uncharacterized protein	Solyc03g062660.2	Solyc03g062660

K4BIL0	Uncharacterized protein	Solyc03g093330.2	Solyc03g093330
K4BJF2	Uncharacterized protein	Solyc03g097350.2	Solyc03g097350
K4BJP3	Uncharacterized protein	Solyc03g098280.2	Solyc03g098280
K4BKB1	Uncharacterized protein	Solyc03g112580.2	Solyc03g112580
K4BKB6	Uncharacterized protein	Solyc03g112630.2	Solyc03g112630
K4BL60	Uncharacterized protein	Solyc03g115580.2	Solyc03g115580
K4BL63	Uncharacterized protein	Solyc03g115610.2	Solyc03g115610
K4BME9	Uncharacterized protein	Solyc03g120000.2	Solyc03g120000
K4BNZ9	Uncharacterized protein	Solyc04g008430.1	Solyc04g008430
K4BP07	Uncharacterized protein	Solyc04g008510.2	Solyc04g008510
K4BP60	Uncharacterized protein	Solyc04g009040.2	Solyc04g009040
K4BPF0	Uncharacterized protein	Solyc04g009950.2;cdc5	Solyc04g009950
K4BPF4	Uncharacterized protein	Solyc04g009990.2	Solyc04g009990
K4BPR1	Uncharacterized protein	Solyc04g012090.1	Solyc04g012090
K4BQD7	Uncharacterized protein	Solyc04g016370.2	Solyc04g016370
K4BRY9	Uncharacterized protein	Solyc04g050170.2	Solyc04g050170
K4BSH7	Uncharacterized protein	Solyc04g054200.2	Solyc04g054200
K4BT85	Uncharacterized protein	Solyc04g064940.2	Solyc04g064940
K4BUD8	Uncharacterized protein	Solyc04g077010.2	Solyc04g077010
K4BV24	Uncharacterized protein	Solyc04g079400.2	Solyc04g079400
K4BVL4	Uncharacterized protein	Solyc04g081350.2	Solyc04g081350
K4BWQ1	Uncharacterized protein	Solyc05g007250.2	Solyc05g007250
K4BZT9	Uncharacterized protein	Solyc05g025510.2	Solyc05g025510
K4C1J5	Uncharacterized protein	Solyc05g051640.2	Solyc05g051640
K4C1R6	Uncharacterized protein	Solyc05g052350.2	Solyc05g052350
K4C1Z3	Uncharacterized protein	Solyc05g053130.2	Solyc05g053130
K4C386	Uncharacterized protein	Solyc06g006040.1	Solyc06g006040
K4C3M9	Uncharacterized protein	Solyc06g008490.2	Solyc06g008490
K4C3R8	Uncharacterized protein	Solyc06g008880.2	Solyc06g008880
K4C4X7	Uncharacterized protein	Solyc06g036080.2	Solyc06g036080
K4C570	Uncharacterized protein	Solyc06g043170.2	Solyc06g043170
K4C5A9	Uncharacterized protein	Solyc06g048620.2	Solyc06g048620
K4C5E1	Uncharacterized protein	Solyc06g048950.2	Solyc06g048950
K4C5K0	Uncharacterized protein	Solyc06g050560.2	Solyc06g050560
K4C7F5	Uncharacterized protein	Solyc06g065260.2	Solyc06g065260
K4C8D8	Uncharacterized protein	Solyc06g069650.2	Solyc06g069650
K4C8V0	Argonaute1-1;AGO1A	AGO1-1;Solyc06g072300.2	Solyc06g072300
K4C935	Uncharacterized protein	Solyc06g073150.2	Solyc06g073150
K4C9B9	Uncharacterized protein	Solyc06g074010.2	Solyc06g074010
K4C9J1	AGO5	Solyc06g074730.2	Solyc06g074730
K4CA49	Uncharacterized protein	Solyc06g076910.1	Solyc06g076910
K4CAX3	Uncharacterized protein	Solyc07g005010.2	Solyc07g005010
K4CBS4	Uncharacterized protein	Solyc07g008050.2	Solyc07g008050
K4CDV6	Uncharacterized protein	Solyc07g040790.2	Solyc07g040790

K4CDY3	Uncharacterized protein	Solyc07g041080.2	Solyc07g041080
K4CEP3	Uncharacterized protein	Solyc07g044850.2	Solyc07g044850
K4CFQ5	Uncharacterized protein	Solyc07g053600.2	Solyc07g053600
K4CH92	Uncharacterized protein	Solyc07g064090.2;FIE	Solyc07g064090
K4CHR2	Uncharacterized protein	Solyc07g065860.2	Solyc07g065860
K4CIU6	Uncharacterized protein	Solyc08g008120.2	Solyc08g008120
K4CIY5	Uncharacterized protein	Solyc08g008510.2	Solyc08g008510
K4CLK9	Uncharacterized protein	Solyc08g066320.2	Solyc08g066320
K4CLM5	Uncharacterized protein	Solyc08g066490.2	Solyc08g066490
K4CLT0	Uncharacterized protein	Solyc08g067040.2	Solyc08g067040
K4CMF7	Uncharacterized protein	Solyc08g074370.2	Solyc08g074370
K4CND9	Uncharacterized protein	Solyc08g077800.2	Solyc08g077800
K4CPB8	Uncharacterized protein	Solyc08g081210.2	Solyc08g081210
K4CPK1	Uncharacterized protein	Solyc08g082050.1	Solyc08g082050
K4CPU3	Uncharacterized protein	Solyc08g082970.2	Solyc08g082970
K4CQA1	Uncharacterized protein	Solyc09g007110.2	Solyc09g007110
K4CSL8	Uncharacterized protein	Solyc09g031610.2	Solyc09g031610
K4CU24	Uncharacterized protein	Solyc09g061940.1	Solyc09g061940
K4CU80	Uncharacterized protein	Solyc09g064520.2	Solyc09g064520
K4CVQ9	Uncharacterized protein	Solyc09g082830.2	Solyc09g082830
K4CWC8	Uncharacterized protein	Solyc09g091020.2	Solyc09g091020
K4CWG6	Uncharacterized protein	Solyc09g091400.2	Solyc09g091400
K4CWZ9	Uncharacterized protein	Solyc09g098290.2	Solyc09g098290
K4CZW5	Uncharacterized protein	Solyc10g047000.1	Solyc10g047000
K4D312	DNA polymerase	Solyc10g081250.1	Solyc10g081250
K4D3U6	Uncharacterized protein	Solyc10g085120.1	Solyc10g085120
K4D603	Uncharacterized protein	Solyc11g011980.1	Solyc11g011980
K4D6L1	Eukaryotic translation initiation factor 3;eIF3i	Solyc11g017070.1	Solyc11g017070
K4D6N3	Uncharacterized protein	Solyc11g017300.1;LOC543616	Solyc11g017300
K4D6Y0	Uncharacterized protein	Solyc11g020280.1	Solyc11g020280
K4DA37	Uncharacterized protein	Solyc11g068800.1	Solyc11g068800
K4DBD3	Uncharacterized protein	COP1;Solyc12g005950.1	Solyc12g005950
K4DBL6	Uncharacterized protein	Solyc12g006790.1	Solyc12g006790
K4DBZ1	Uncharacterized protein	Solyc12g009030.1	Solyc12g009030
K4DG04	Uncharacterized protein	Solyc12g056730.1	Solyc12g056730
K4DGW5	Uncharacterized protein	Solyc12g088940.1	Solyc12g088940
K4DH65	Uncharacterized protein	Solyc12g095920.1	Solyc12g095920
O22466	WD-40 repeat-containing protein MSI1	MSI1	Solyc01g104510
Q6RFY3	MAP3Ka	LOC543918;Solyc11g006000.1	Solyc11g006000
Q8GUQ5	tBRI1;Altered brassinolide sensitivity 1;Brassinosteroid LRR receptor kinase;	CURL3	Solyc04g051510
Q9ZNU6	tDET1;Deetiolated1 homolog;hp2; DET1	dg;hp2;DET1	Solyc01g056340

Table S7. The coding sequences, primers, and restriction sites (RS) used for Y2H assays.

Sol ID	Outer Primers(L)	Internal Primers(L)	Internal Primers(R)	Outer Primers(R)	RS (L)	RS(R)
Solyc02g021470	——	ATGAAGAAAGCTAAGTCAC	——	CTAAGCAAGGTAGTTGTATA	GAATTC	CTCGAG
Solyc02g021650	——	ATGAGTGTATGGAACTACGTGG	——	CTAATGCAACCTTGTCAACTC	GAATTC	GGGCCC
Solyc01g090750	TTCCCAACATTCTCATCACT	TAGTCGGAAATGGCGTAT	TTAGGACAAGTCCAGGTCTAT	GCTGCCTAATCCTACTCATTCCAGGTCTAT	CCGCGG	GGGCCC
Solyc02g090430	AGAAGGAAAGGGAGGATT	AACTACCACAGGCAATGC	GATTAAATGCCGACAGGA	GAGCTACCTCTCTAGCGATAA	GAATTC	CTCGAG
Solyc03g117630	ACCGCCACAATGTCTTA	TTTACTAATGGCGAAATCTG	CTCAAAGCAGTCACTAAAATA	TCTTTAATCAACTTCCTCAAT	GAATTC	CTCGAG
Solyc04g015130	ACTTATTCATCTGTATCATTG	TCACTGATGGCTTCGAATT	TACCGTTTAGACATCCTTGTA	AGTCGGGCTGATAATTACTAGAAG	CCGCGG	GGGCCC
Solyc05g005930	GCTCCTCTTTCATCACTCT	TAAAGCATACAATGAGTACTAC	TTACGATAAGTTCAATGACAA	TTCACTTATTACTATTCCTACA	CCGCGG	GGGCCC
Solyc05g018570	AGCAGATAAATGAAAGGGTT	AATGGCGTCAGCTGATGTTGA	TACTAAAGCCGCAGGAAA	ATCGACCATCATCTACTTCCACAG	CCGCGG	GGGCCC
Solyc06g072040	AGAGCACCATTACACCTT	ATGGACAACAACCCTCACC	AAGGCAACAAGATAACATAG	AGCAGAAACGACAAACTTACA	GAATTC	CTCGAG
Solyc06g074780	CGACAGATCAAATCCCTATA	AACAATGGCACCAAAGAC	GAGATTCATTAGCATTCATATT	AAGCCCTAATTGCTAGTAAA	GAATTC	CTCGAG
Solyc06g082440	TAGCCAATTCAAAGACCAT	ATCCATTTACCGATGCC	AAATAAAATCACCCAATGC	ATGCTTGAAATTTAGCTGTC	GGGATC	GGGCCC
Solyc07g065840	TTCCATTTCGCCTTACAG	AAATGTCGGACGTAGAGACGT	GTTCTGATGGGATAAAGCA	GAACTTAATCAACCTCCTCCA	CCGCGG	GGGCCC
Solyc09g010630	TTCCTCCTTACAAACCCTA	AGTAGTAGATATGGCTGGAA	ATGAGGATTGAGGAGCGA	AAACAGCTTAGTCGACC	CCGCGG	GGGCCC
Solyc09g018730	TCTGATGATCTCCCTGTTTC	GCAGCAGATGCAAGTAAA	TTGCCTTCAACAAGAACTAA	TTCATCTAGGATTGGTGGT	CCGCGG	GGGCCC
Solyc09g092500	CATAATACTA	TTTAAGTTTGT	TTGGTTGTATG	TCAACAACT	CCGC	GGGC

	ATTGACCTCGAA	GAGTGTAAA	CCATATTTT	TTTGCTAACT	GG	CC
Solyc10g083760	ATTTACATCTCCGGCTAC	AATGGAGGTTCTTCGGTTTA	CCAGAACAAGCCATAACA	TCAATGCATTATGAGCTGGT	CCGCGG	GGGCCC
Solyc11g007480	TAAATAAGGTAGCCAAGAC	CAAGAGCTTGATCATGG	GACCATCAACAAGAACAT	CACATACAATATAATTATGTTAAT	CCGCGG	GGGCCC
Solyc11g069700	TGGCTACCTAAACTCGAT	GTATTTGACATTTGTTTCTATTG	CAGTTCTGGGAAAGGATA	AGTTGAACTGCACTATTCATT	CCGCGG	GGGCCC
Solyc11g070040	AAGATCGAATGGTAACTCCT	TTTCTTTCTACAAGGAGGAT	TCAGATTCAACAACAGCAGA	TTCTTAATCAGATAAGGGTTC	CCGCGG	GGGCCC
Solyc12g057060	AACAATCCTTCATTTCATTTC	TTATGAAACAATGGGTTCAA	TCCGGTACTTCATACAACAA	ATTTTGACTTAATGCTTGGAT	CCGCGG	GGGCCC

Table S8. The coding sequences, primers, and restriction sites (RS) used for BiFC analysis.

Sol ID	Primers(L)	Primers(R)	RS (L)	RS (R)
Solyc02g021470	ATGAAGAAAGCTAAGTCAC	CTAAGCAAGGTAGTTGTATA	GTCGAC	GTCGAC
Solyc02g021650	ATGAGTGTATGGAACTACGTGG	CTAATGCAACCTTGTCAACTC	GTCGAC	GTCGAC
Solyc01g090750	TAGTCGGAAATGGCGTAT	GCTGCCTAATCCTACTCAT	CCCCGGG	TCTAGA
Solyc02g090430	AACTACCACAGGCAATGC	GAGCTACCTCTCTAGCGATAA	CCCCGGG	TCTAGA
Solyc06g082440	ATCCATTTACCGATGCC	ATGCTTGAAATTTAGCTGTC	CCCCGGG	TCTAGA
Solyc09g092500	TTTAAGTTTGTGAGTGTAAA	TCAACAACTTTTGCTAACT	CCCCGGG	AAGGCCT
Solyc11g007480	CAAGAGCTTGATCATGG	CACATACAATATAATTATGTTAAT	CCCCGGG	TCTAGA
Solyc11g069700	GTATTTGACATTTGTTTCTATTG	AGTTGAACTGCACTATTCATT	CCCCGGG	TCTAGA
